# Label-free quantitative proteomics of maize roots from different root zones provides insight into proteins associated with enhance water uptake

**DOI:** 10.1186/s12864-022-08394-y

**Published:** 2022-03-06

**Authors:** Junqiao Song, Daowen Lu, Yongfeng Niu, Haichao Sun, Pan Zhang, Wenheng Dong, Yongjiang Li, Yingying Zhang, Lianyong Lu, Qi Men, Xiaohui Zhang, Pengxun Ren, Chuankui Chen

**Affiliations:** 1 College of Agronomy, Henan University of Science and Technology, Luoyang, China; 2 Maize Research Institute, Anyang Academy of Agricultural Sciences, Anyang, China; 3 Hebei Runnong Water Saving Technology Co., Ltd., Tangshan, China

**Keywords:** Maize, Roots, irrigation, Field capacity, Root zones, Label-free proteomics, Water uptake, Soil layers

## Abstract

**Background:**

Maize is one of the most important food crops worldwide. Roots play important role in maize productivity through water and nutrient uptake from the soil. Improving maize root traits for efficient water uptake will help to optimize irrigation and contribute to sustainable maize production. Therefore, we investigated the protein profiles of maize cv. Anyu308 root system divided into Upper root zone (UR), Middle root (MR), and Lower root (LR), by label free quantitative shotgun proteomic approach (LFQ). The aim of our study was to identify proteins and mechanisms associated with enhanced water uptake in different maize root zones under automatic irrigation system.

**Results:**

At field capacity, MR had the highest water uptake than the UR and LR. We identified a total of 489 differentially abundant proteins (DAPs) by pairwise comparison of MR, LR, and UR. Cluster analysis of DAPs revealed MR and UR had similar protein abundance patterns different from LR. More proteins were differentially abundant in MR/UR compared to LR/MR and LR/UR. Comparisons of protein profiles indicate that the DAPs in MR increased in abundance, compared to UR and LR which had more downregulated DAPs. The abundance patterns, functional category, and pathway enrichment analyses highlight chromatin structure and dynamics, ribosomal structures, polysaccharide metabolism, energy metabolism and transport, induction of water channels, inorganic ion transport, intracellular trafficking, and vesicular transport, and posttranslational modification as primary biological processes related to enhanced root water uptake in maize. Specifically, the abundance of histones, ribosomal proteins, and aquaporins, including mitochondrion electron transport proteins and the TCA cycle, underpinned MR’s enhanced water uptake. Furthermore, proteins involved in folding and vascular transport supported the radial transport of solute across cell membranes in UR and MR. Parallel reaction monitoring analysis was used to confirmed profile of the DAPs obtained by LFQ-based proteomics.

**Conclusion:**

The list of differentially abundant proteins identified in MR are interesting candidates for further elucidation of their role in enhanced water uptake in maize root. Overall, the current results provided an insight into the mechanisms of maize root water uptake.

**Supplementary Information:**

The online version contains supplementary material available at 10.1186/s12864-022-08394-y.

## Introduction

Globally, agriculture is the largest freshwater user [1]. In China irrigated agriculture accounts for 62% of the country’s total gross water withdrawal, and 84% of net water abstraction [2, 3]. Further groundwater withdrawal is unsustainable due to demand on the available water resources from non-agricultural uses, rise in population, and urbanization [4–7]. To increase crop production with limited water resources requires developing new cultivars with enhanced root water and nutrient uptake, especially in areas where agricultural sustainability is threatened by the continuous decline of the regional groundwater Table [7].

Maize (*Zea mays* L.) is one of the most important cereal crops in China and accounts for about 40% of the country’s cropland for cereal crops, particularly in the North and Northeast region; where groundwater is the major source of irrigation [2, 8, 9]. Maize is a critical feedstock for animal and ethanol production in China, and water demand for maize production is expected to increase in the future. Therefore, identifying root traits that can increase the ability of the maize root system to extract water more efficiently from the soil can help optimize and save irrigation water in maize production systems.

Roots play an essential role in plant water acquisition from the soil and are the gate-way for plant water and nutrient supply [2, 10–15]. Root water uptake is used to optimize irrigation and fertilizer application [13, 15]. Generally, water uptake by roots is primarily controlled by root system distribution, stomatal conductance, water channels, hydraulic conductivities of root types, water availability, and soil properties, genetics, and climatic conditions [15–19]. Overall, root water uptake is crucial to maize productivity, however, despite its importance, little information is available on the protein profile underlaying root-type soil water acquisition through the root zones and its radial movement in maize roots.

Previous studies described the developmental processes associated with maize root architecture [20–23], and other studies have employed mathematical models [24–28], isotope analysis [29–31], and molecular approaches [32–34] to better understand maize root distribution and water uptake patterns from the soil in different environments. These studies revealed that maize roots, i.e., primary, seminal, and lateral roots cover more than 80% of the total root lengths distributed mainly near the subsurface top 45 cm soil layer and supply about 65% of the total water requirement [18, 20, 21]. Additionally, lateral roots play significant role in water and nutrient uptake in the seedling stage, and the crown roots maintain water uptake in the matured maize root system [20]. Recently, the longitudinal structure of maize roots was shown to consist of distinct zones, i.e., meristematic, elongation, and differentiation root zones with functionally distinct cell types, contributing to the differences in hydraulic conductivities among root types [15, 33]. Similarly, maize root water channels (aquaporins) mediate internal water potential by abundance of ions and solutes, contributing to the variability in water uptake of root at different root zones [33–35], and aquaporins are known to be under a dynamic biochemical and metabolic control [29–35]. Therefore, understanding protein associations and biochemical mechanisms that underpin maize root water acquisition through the root zones could help identify important root trait for maize improvement.

Proteomic studies in the post genomic era are helping the understanding of protein functions in plant physiology across different conditions. For instance, label-free quantitative proteomics (LFQ) technique offers high resolution and deep coverage of protein abundance in whole and specific root tissues and provides an easy and powerful way for identifying and quantifying thousands of proteins from a complex biological sample. This method has been demonstrated in many studies that identify functional and structural proteins involved in shaping the three-dimensional architecture maize root system [36–39], including root type-specific proteins in maize primary root [33], study maize embryo proteome [37] and maize root development in response to salt stress [38].

In this study, we used the LFQ-based proteomics method to identify proteins and quantify their abundance in relation to water uptake by different maize root zones i.e., UR, MR and LR respectively under automatic irrigation. Our result indicate that MR had the most efficient water uptake under field capacity, possibly due to the differentially abundance of proteins functionally associated with chromatin modification, energy metabolism and electron transport chain, inorganic ion transport, intracellular trafficking, and vesicular transport.

## Results

### Maize root distribution and water uptake at different soil layers

**Fig. 1:**
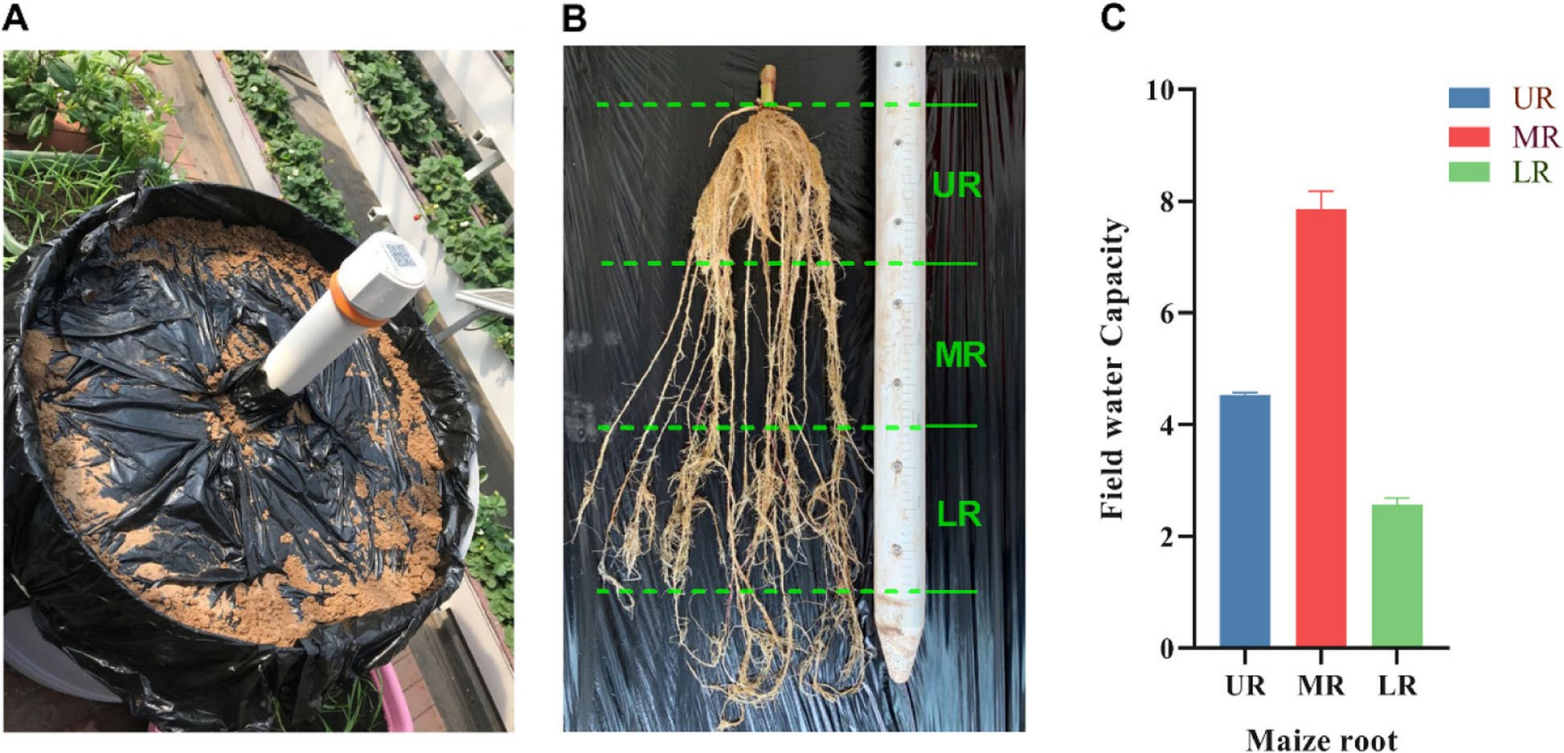
Root growth and water uptake by maize roots. **a** Experimental set up with multi-depth soil smart moisture meter, **b** Maize root length **c**. Variation in water uptake by different maize roots in the soil. Values are means ±SD (*n* = 3 biological replicates). Statistical significance was analyzed using the two-sided Student t-test. The asterisk indicates a significant difference (* *p* < 0.05)

As shown in Fig. 1a-c, the upper root, measured from 0 to 20 cm (UR), middle root, 20-40 cm (MR), lower root 40-60 cm (LR) (Fig. 1b). The soil water potentials for UR, MR, and LR were 4.4 g cm, 7.9 g cm, and 2.5 g cm, respectively (Fig. 1c). The comparison of the root water uptake between the three zones showed that MR had the most efficient water extraction rate from the soil, followed by UR, and LR respectively (Fig. 1c).

### Protein identification

**Fig. 2:**
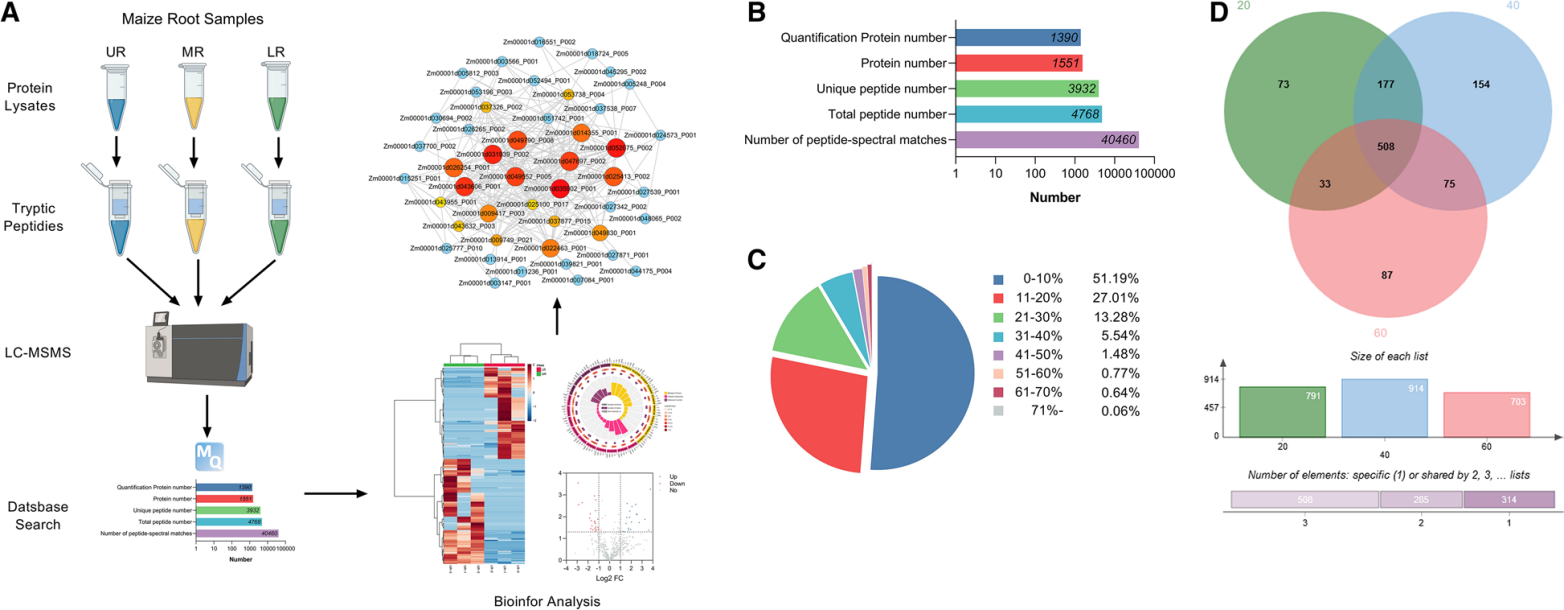
Experimental work flow and LFQ proteins identification different maize root zones. **a** The LFQ workflow showing protein extraction from the three root samples UR, MR, and LR and LC-MS/MS based protein identification and quantification. **b** Protein sequencing statistics. Blue, red, green, sky-blue, purple bars represent quantified protein number, protein number, unique peptide number, total peptide number, number of peptide spectral matches. **c** Protein coverage. **d** Venn diagram showing number of common and unique proteins identified in UR, MR and LR respectively

In this study we employed the robust LFQ based quantitative proteomics technology to establish the proteomic changes underpinning the enhanced root water uptake observed in MR. The experimental workflow for the LFQ based quantitative proteomics is shown in Fig. 2. Proteins identified in the three biological replicates at 1% FDR, were used for further analysis. For example, the mass spectrum interpretation allowed us to identify 1551 proteins and quantify 1390 proteins from 40,460 peptides spectral matches (Fig. 2b) covering more than 71% of the total proteins in each sample (Fig. 2c, Additional file 1: Table S1, Additional file 2: Table S2). The correlation plots for three biological replicates of all the samples reported in Additional file 3: Fig. S1, indicate a high reproducibility of the proteins (R2 ≥ 0.95) identified in the replicate of each sample. As shown in Fig. 2d, a total of 791 proteins (72% of 1107) were identified in UR, 914 (83% of 1107) in MR, and 703 (64% of 1107) in LR respectively, which indicates that 11 and 19% more proteins were abundant in MR compared to UR and LR, which supports the need for more water-uptake as seen in MR (Fig. 1). Furthermore, the samples shared a subset of 508 (46% of 1107) abundant proteins whereas 72, 154, and 87 proteins were uniquely abundant in UR, MR, and LR respectively (Fig. 2d). Overall, the results suggests that more proteins were abundant in MR and the dynamic changes in MR protein abundance may contribute to its improved water uptake.

### Identification of differentially abundant proteins in the three root zones

**Fig. 3:**
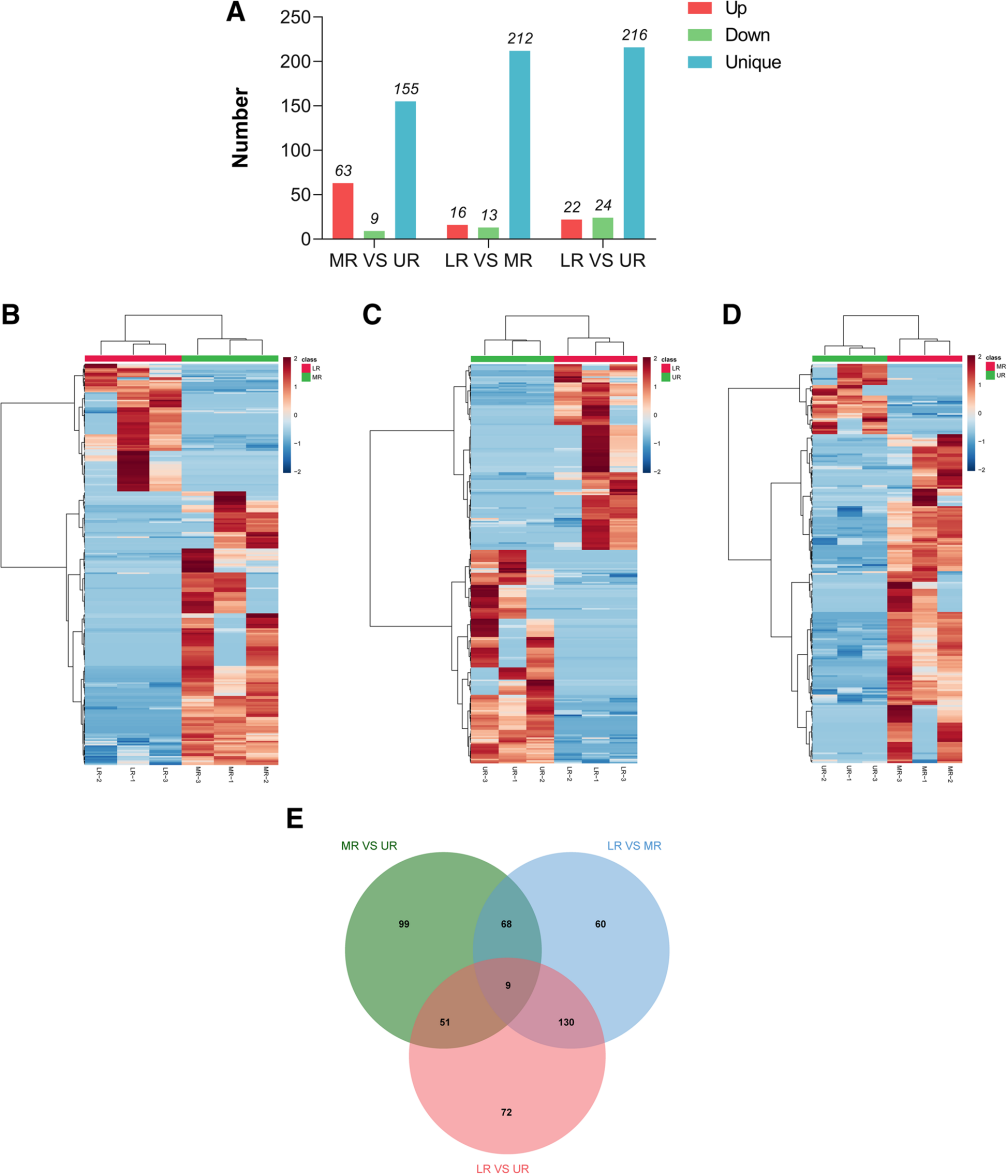
Number of DAPs and their profile in the three maize root zones. **a** bar chart displaying the number of up-regulated and down-regulated proteins in each pairwise comparison of MR/UR, LR/MR, and LR/UR. Red, green, and blue bars represent up-regulated down-regulated and unique DAPs. **b**, **c**, **d** Heat map showing abundance profile of proteins in MR/UR, LR/MR, and LR/UR comparison. Proteins with high abundance (brown); proteins with low abundance (dark brown) of what is contained in the second panel. **e** Venn diagram showing unique and common DAPs

A total of 489 proteins were differentially abundant (DAPs) based on pairwise comparison of all the quantified proteins in three samples (MR/UR, LR/MR and LR/UR, Fig. 3a-e, Additional file 4: Table S3). DAPs greater than 2.0-fold and 0.50-fold (*P*-value ≤0.05) were grouped as significantly up-regulated and down-regulated respectively in each comparison. Further analysis revealed that MR/UR and LR/MR comparisons had similar abundance profiles different from LR/UR (Fig. 3b-d) which suggests that MR and UR might have similar regulation of cellular functions in their overall root proteome opposite to LR. Furthermore 227 DAPs were identified in MR/UR comparison, including 63 up-regulated, 9 downregulated and 155 specific DAPs (Fig. 3a and Additional file 4: Table S3). The LR/MR had 267 DAPs, of which 23 DAPs were up-regulated, 32 DAPs were downregulated and 212 DAPs were unique (Fig. 3a and Additional file 4: Table S3). Similarly, LR/UR had 262 DAPs, including 22 up-regulated, 24 downregulated and 216 specific DAPs (Fig. 3a and Additional file 4: Table S3). Here, more DAPs were down-regulated in LR/MR and LR/UR compared to MR/UR which had more up-regulated DAPs, further supporting our findings that more proteins were positively induced in MR to that underlay the enhanced rates of water uptake in MR.

Data set overlap revealed 9 commonly shared DAPs between the three comparisons (Fig. 3e). Meanwhile, 68 DAPs were shared between MR/UR and LR/MR, and 51 DAPs between MR/UR and LR/UR, and 130 DAPs were common between LR/MR and MR/UR (Fig. 3e). In contrast a total of 99, 60, and 72 DAPs were unique to MR/UR, LR/MR, and LR/UR, respectively (Fig. 3e).

### Gene ontology and enrichment analysis of differentially regulated proteins

To reveal proteins in maize root zones (UR, MR and LR) that may be involved in enhanced water-uptake, we classified the DAPs with Gene ontology (GO) classification and functional enrichment of GO categories.

**Fig. 4:**
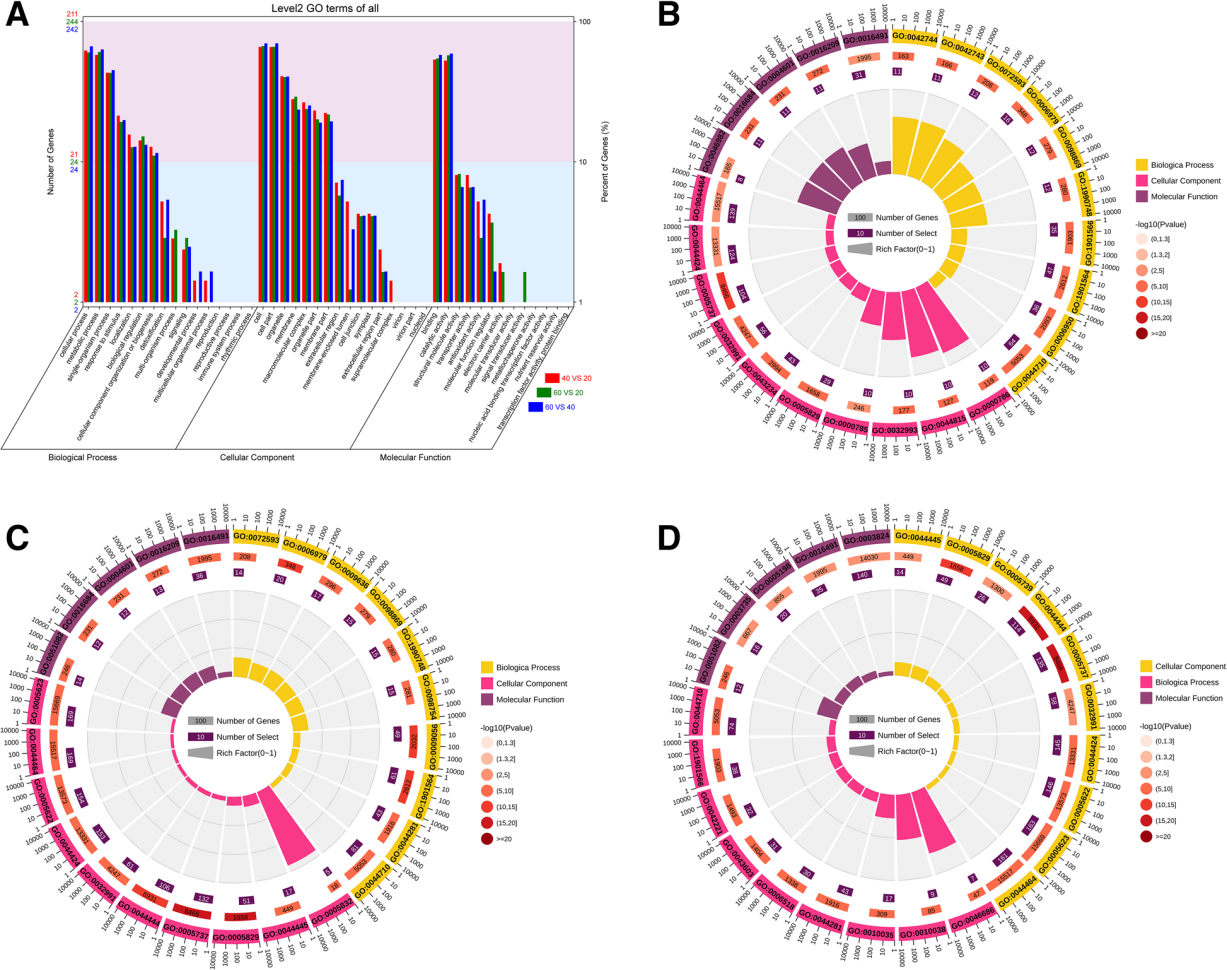
Gene ontology classification and enrichment analysis of DAPs. **a** Histogram of major GO term: biological process, cellular component, molecular function. The blue bar represents biological process categories, the red bar represents GO terms for cellular component, and the yellow bar represents biological process categories to molecular function categories. **b** Enrichment of GO terms in each comparison MR/UR comparison (**c**) LR/MR comparison (**d**) LR/UR comparison. Orange, pink and purple color represents enriched biological process, cellular component, molecular function GO terms

The DAPs were grouped into three GO terms, i.e., biological process, cellular component, and molecular function. The distribution plot of GO terms revealed a total of 45 GO terms among the three root zones (Fig. 4 and Additional file 5: Table S4). Furthermore, the DAPs were subcategorized into 16 main hierarchically structured GO classifications including 16 biological processes (BP), 16 molecular functions (MF), and 13 cellular components (CC) (Fig. 4a).

GO enrichment analysis revealed significantly over-represented biological process, cellular components, and molecular functions category (*p* < 0.05) (Fig. 4, Additional file 5: Table S4). For MR/UR the top enriched BP include “tricarboxylic acid biosynthetic process,” “hydrogen ion transport”, and “organonitrogen compound metabolic process.” Also, there was significant up-regulation of “peptide metabolic process,” “oxidation-reduction process,” “response to pH,” and “cell wall organization” (Fig. 4b). The induction of the top BPs suggests abundance of enzymes that are involved in providing energy and carbon skeleton for root cellular functions and could contribute to water uptake in MR. Meanwhile, in LR/MR, the most abundant enriched BP were “organonitrogen compound metabolic process,” “response to stimulus,” “pyruvate metabolic process,” “cellular amide metabolic process,” “cellular aldehyde metabolic process,” and “ADP metabolic process” (Fig. 4c). Further analysis indicate that DAPs involved in response to stimulus, and aldehyde metabolism were positively induced whereas pyruvate metabolism were repressed. In LR/UR, the top enriched BP include “pyruvate metabolism,” “organonitrogen compound metabolic process,” “small molecule metabolic process, and “response to metal ion,” (Fig. 4d). (Additional file 5: Table S4).

### Clusters of orthologous groups (COG) functional classification

**Fig. 5:**
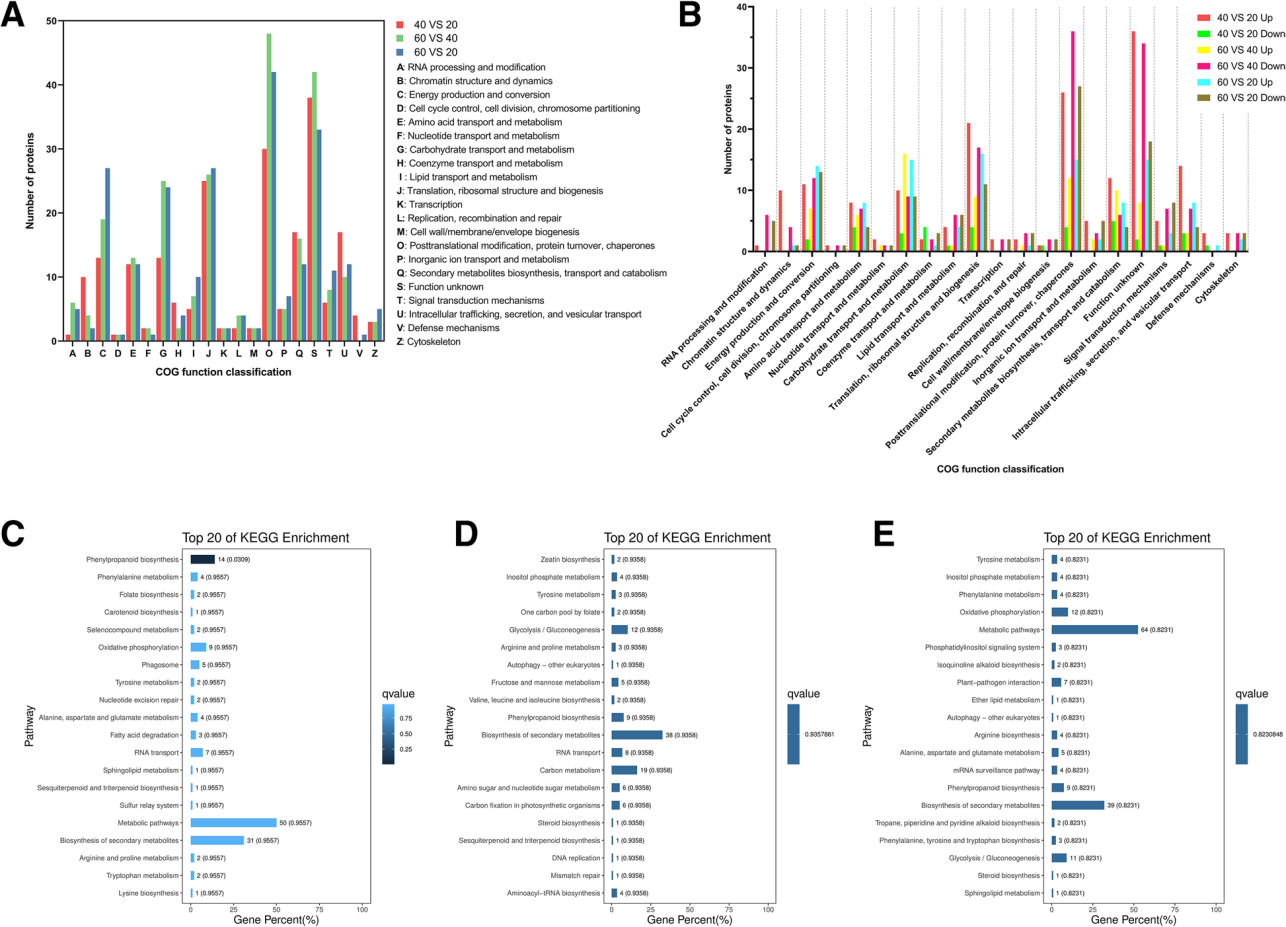
COG classification and KEGG pathway enrichment for differentially abundant proteins. **a** The number of functional proteins in each COG category. Red, green, and blue bars represent DAPs in MR/UR, LR/MR, and LR/UR. **b** Differential regulation of COG categories in MR/UR, LR/MR, and LR/UR, red, green, yellow represent up-regulated and light-red, turquoise, dark bars represent down-regulated DAPs MR/UR, LR/MR, and LR/UR, respectively. KEGG pathways enrichment analysis (**c**) MR/UR comparison. **d** LR/MR comparison. **e** LR/UR comparison

COG analysis was used to further characterize the DAPs that might be involved maize root water-uptake in the different root zones. The COG distribution plot revealed that MR/UR, LR/MR, and LR/UR had 21, 20, and 21 COG categories respectively (Fig. 5, Additional file 6: Table S5). The significantly abundant COG categories in the three comparisons were category C (Energy production and conversion); G (Carbohydrate transport and metabolism); J (translation, ribosomal structure, and biogenesis); O (Posttranslational modification, protein turnover, chaperones); Q (Secondary metabolites biosynthesis, transport; and catabolism); and S (functions unknown) (Fig. 5a). Further analysis showed that the DAPs in category B, C, and P were up-regulated in MR/UR compared to LR/MR. These proteins are primarily involved in energy production and ion transport which have function in solute uptake in root hair cells. In LR/MR, 75% of DAPs in the O category were highly repressed, in contrast to their up-regulated profile in MR/UR. The same trend was observed in COG category J and U in which the DAPs participate in protein translation, ribosome and cellular transport and could contributed to root water uptake and transport. Nevertheless, we observed that DAPs in E and G categories were consistently up-regulated proteins in the three-comparison group (Fig. 5b, Additional file 6: Table S5) suggesting that DAPs in this category is important for root functions including water acquisition from the soil. These results suggest that DAPs in COG categories B, C, E, G, J, P, and U might play a significant role in maize root water uptake.

### KEGG pathway analysis of DAPs

In MR/UR, KEGG pathway analysis (Fig. 5c-e, and Additional file 7: Table S6), revealed the enrichment of carbohydrate metabolism consisting primarily of DAPs related to glycolysis, pyruvate biosynthesis, and D-glyceraldehyde 3-phosphate, AMP biosynthesis and tricarboxylic acid cycle pathway. Other significantly enriched pathways within this group include biosynthesis of secondary metabolites, amino acid metabolism, and metabolism of cofactors, (Fig. 5c). In the LR/MR, starch biosynthesis, N-glycan metabolism, amino-acid degradation, terpenoids, polyketide metabolism, protein modification, and energy metabolism were significantly enriched (Fig. 5d). In LR/UR, amino acid metabolism, lipid metabolism, and carbohydrate metabolism were the most enriched pathway (Fig. 5e). Overall, the KEGG highlighted the abundance of different pathway active in different root zones which underscored the complex nature of root cellular functions.

### Profiles of DAPs associated with water uptake in the different root zones

#### Profile of DAPs in MR/UR

As stated earlier, overlapping the LFQ data of the three comparisons revealed that in the MR/UR group, 72 DAPs showed significant differences in abundance of which 63 DAPs were up-regulated, and 9 DAPs were down-regulated (Fig. 3 and Additional file 4: Table S3). Among the up-regulated proteins, we noticed high abundance of proteins that played a role in chromatin structure and DNA repair. They include seven histones with an average of 9 foldchange. At the same time several ribosomal proteins were significantly up-regulated, as well as enzymes of the tricarboxylic acid cycle for example, succinate dehydrogenase (Zm00001d018758 _P004, FC = 2.0), succinate-CoA ligase [ADP-forming] (Zm00001d006667 _P001, FC = 2.8), mitochondrial ATP synthase subunit (Zm00001d053983 _P003, FC = 2.1), and alcohol dehydrogenase 1 (ADH). (Zm00001d033931 _P002, FC = 5.2). Studies suggests that histones are subject to numerous covalent modifications and these modifications control many aspects of chromatin function including histone mediated gene expression [40, 41]. Increase in abundance of ribosomal proteins has been reported in maize seminal roots during drought [42] and the TCA cycle generate cellular energy metabolism and carbon skeleton supply for root development [43], likely contributed to the enhanced water uptake observed in MR.

Within this group, proteins involved in antioxidant system and defense response were highly up-regulated such as glutathione transferase11 (Zm00001d027539 _P001, FC = 11), uncharacterized protein (Zm00001d016408 _P001, FC = 8.8) containing CYSTM domain, ABA-responsive protein (Zm00001d023664 _P001, FC = 8.4), stress-responsive protein (Zm00001d022420 _P001, FC = 6), and pathogenesis-related (PR) protein 10 (Zm00001d028815 _P001, FC = 17). We also noticed up-regulation of water ions and versicular transport system. They include aquaporin PIP1-5 (Zm00001d051872 _P001, FC = 2.7) hydrogen-transporting ATP synthase (Zm00001d011278 _P003, FC = 2.5), and coatomer subunit delta (Zm00001d023943 _P007, FC = 2.3).

Contrastingly, the proteins involved in lipid catabolism: lipoxygenases (Zm00001d042540 _P004, FC = 0.4; Zm00001d042541 _P003, FC = 0.1), and peroxidases: (Zm00001d002901 _P001, FC = 0.4; Zm00001d014467 _P001, FC = 0.2) were the most down-regulated DAPs in MR/UR. The repression of these proteins in MR/UR indicates a reduction in lipid peroxidation in MR which could also favor more water uptake.

#### Profile of DAPs in LR/MR

Figure. 3b show the profiles of DAPs in the LR/MR comparison. Within this group, 23 DAPs were up-regulated, and 33 were down-regulated (Table S3). Pairwise comparison, revealed abundance of DAPs involved in glycolysis and carbohydrate metabolism, they include glyceraldehyde-3-phosphate dehydrogenase 3, cytosolic GAPDH (Zm00001d051001 _P004, FC = 5), pyruvate kinase (Zm00001d001831 _P002, FC = 2.6), pyruvate decarboxylase (Zm00001d028759 _P001, FC = 3.8), Enolase1 (Zm00001d045431 _P002, FC = 2.0), and fructokinase1 (Zm00001d042536 _P001, FC = 4.6). These proteins provide substrate for energy production and conversion within the root cells. Additionally, our result showed abundance of peroxidases. Peroxidases are known to scavenge oxygen radicals that cause oxidative or osmatic stress [44]. Although high levels of ROS can have a negative impact on plant cells, including root cells, however, one study has shown that removal of apoplastic H_2_O_2_ in the maize root growth zone by ROS scavenging differentially increase root cell production and root elongation [45]. Perhaps oxidative stress response is a common phenomenon in maize root zones and may support root development. Other significantly abundant protein within this group, includes endochitinase (Zm00001d018966 _P001, FC = 15), and alcohol dehydrogenase 1(ADH1) (Zm00001d033931 _P002, FC = 14.2). Endochitinase is another defense response protein belonging to the O-Glycosyl hydrolase family which hydrolyze chitin oligosaccharides and the ADH1 is involved in reduction of NAD to NADH thereby supplying reducing power to drive root cellular process.

Interestingly, we detected the induction of another aquaporin isoform: aquaporin PIP2-3 (Zm00001d051174 _P001, FC = 7.3) which is involved in root water uptake. as well as an ion transmembrane transporter H (+)-exporting di-phosphatase (Zm00001d046591 _P003, FC = 2.2), a protein transporter involved in transmembrane ion exchange. It is likely that the induction of these DAPs seems to be important for normal cellular function of the maize root system because similar protein functions was up-regulated in MR.

Among down-regulated proteins were several ribosomal proteins, i. e., 40S ribosomal SA, S3, and S2-1 (Zm00001d048346 _P001, FC = 0.46, Zm00001d024511 _P002, FC = 0.37, Zm00001d013034 _P001, FC = 0.17) and histone proteins in contrast to the up-regulation of their isoform in MR, indicating the histones and ribosomal proteins played important contributes to enhanced water uptake in MR. We also observed the repression of methyl binding domain105 (Zm00001d041290 _P003, FC = 0.32), villin-2 (Zm00001d011854 _P002, FC = 0.32), tubulin alpha chain (Zm00001d006651 _P001, FC = 0.24), and syntaxin-132 (Zm00001d041716 _P006, FC = 0.44). Analysis of the GO biological process indicated that the methyl binding domain protein plays a vital role in the epigenetic control of plant growth and development. The villin-2 is an actin-filament bundle assembly protein, and tubulin alpha chain is involved in microtubule cytoskeleton organization, and syntaxin protein is a major vesicle-mediated transporter playing a significant role in intracellular trafficking and vesicular transport. The significant repression of these DAPs might account for the reduced water uptake in LR.

Furthermore, abscisic acid stress ripening3 (Zm00001d003712 _P001, FC = 0.31), glycine-rich RNA-binding (Zm00001d013568 _P001, FC = 0.24), and putative inactive receptor kinase (Zm00001d044434 _P001, FC = 0.31) were among the proteins that significantly decreased in abundance in LR opposite to their up-regulation in MR. Cysteine synthase (Zm00001d031136 _P003, FC = 0.48), phosphoenolpyruvate carboxylase (Zm00001d020057 _P006, FC = 0.46) and mitochondrial phosphate carrier protein 3 (Zm00001d018187 _P001, FC = 0.28) also decreased significantly in abundance. The decrease in abundance among these DAPs in LR which were up-regulated in MR, especially, those related to protein synthesis, electron transport chain, and chromatin structures, could negatively impact LR water uptake.

### Protein-protein interaction of DAPs

**Fig. 6:**
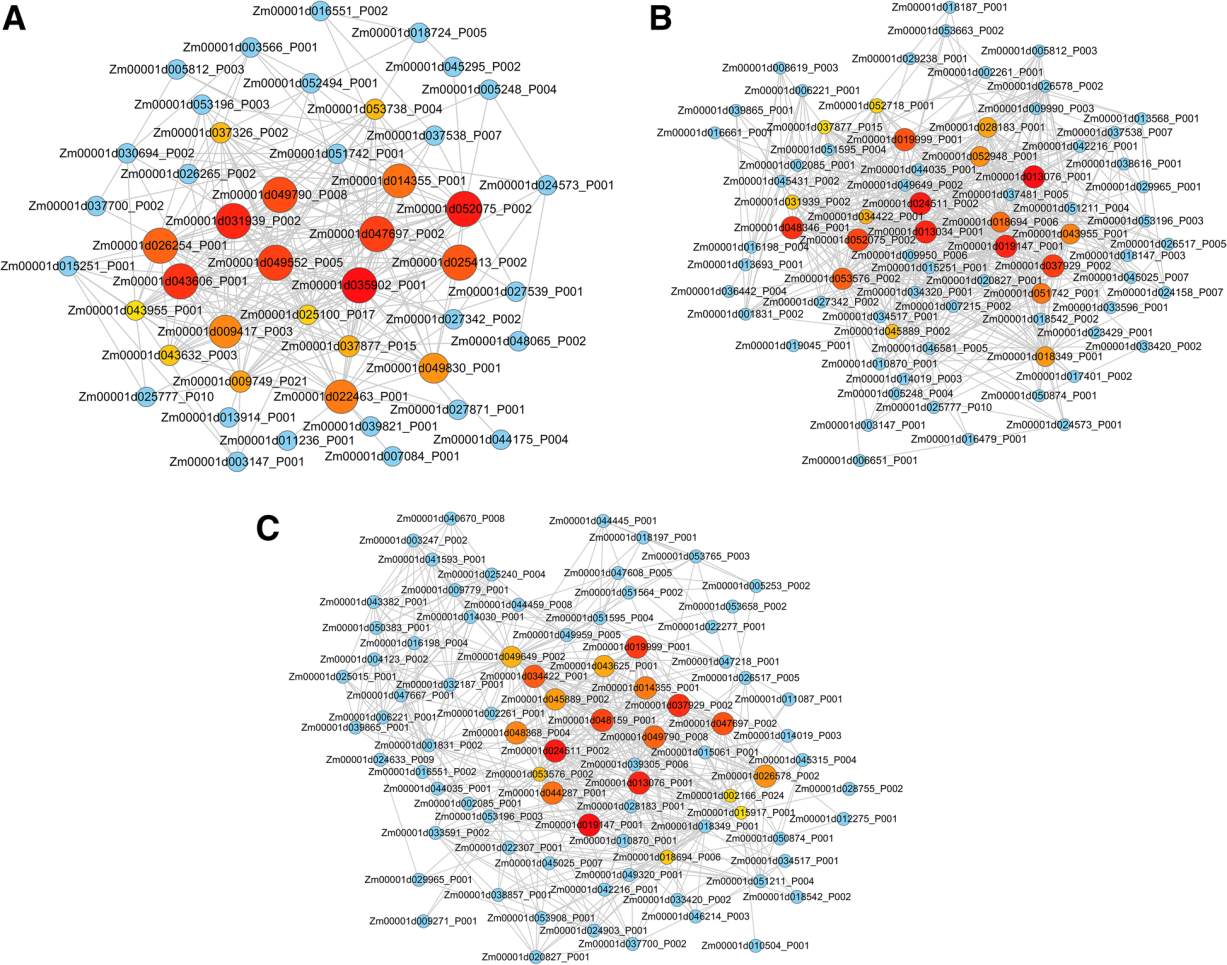
Protein-protein interaction network. **a** Network interaction of differentially regulated proteins in MR/UR. **b** LR/MR. **c** LR/UR. Up-regulated proteins are represented by orange color, and red color represented down-regulated proteins. Unique DAPs are represented by blue color. Big cycle represents hub proteins

To reveal protein association that may be related to enhanced root water uptake, we analyzed the protein-protein interaction (PPI) among DAPs in MR/UR, LR/MR and LR/UR using STRING (http://string-db.org) (Fig. 6, Additional file 8: Table S7). In the PPI network of MR/UR we detected strong interaction among DAPs that acted as hubs within the network (Fig. 6a). The hub proteins include, eukaryotic translation initiation factor 2 eIF-2 (Zm00001d009749 _P021, 21 connections), was associated with macromolecule localization, and many ribosomal proteins: 40S ribosomal protein S28 (Zm00001d047697 _P002, 20 connections), 40S ribosomal protein S25-2 (Zm00001d031939 _P002, 20 connections), and 60S ribosomal protein L22-2 (Zm00001d022463 _P001, 19 connections) were involved in protein translation. In addition to DNA repair protein RAD23-1 (Zm00001d053738 _P004, 15 connections) associated with nucleotide excision repair and T-complex protein 1 subunit delta (Zm00001d051742 _P001, 20 connections) a molecular chaperon involved in protein folding. (Additional file 8: Table S7). Here most of these hub proteins were positively induced and played a significant role in cell wall organization and protein synthesis which could potentially contribute to enhanced water uptake in MR.

In contrast to MR/UR, in LR/MR, we observed strong interaction among hub proteins that significantly down-regulated. These DAPs are involved in glucose metabolism, electron transport chain and energy metabolism. They include pyruvate kinase protein (Zm00001d001831 _P002, 22 connections), glucose-6-phosphate 1-dehydrogenase (Zm00001d025015 _P001, 17 connections), succinate dehydrogenase (Zm00001d049649 _P002, 33 connections) (Fig. 6b, Additional file 8: Table S7). Meanwhile in LR/UR we found hub DAPs involved in ribosomal structures ribosomal proteins (Zm00001d024511, 53 connections; Zm00001d048159, 32 connections; and Zm00001d047697, 69 connections). These proteins were repressed in LR but up-regulated in UR (Fig. 6c, Additional file 8: Table S7).

### Validation of differentially abundant proteins by LC-PRM/MS

**Fig. 7:**
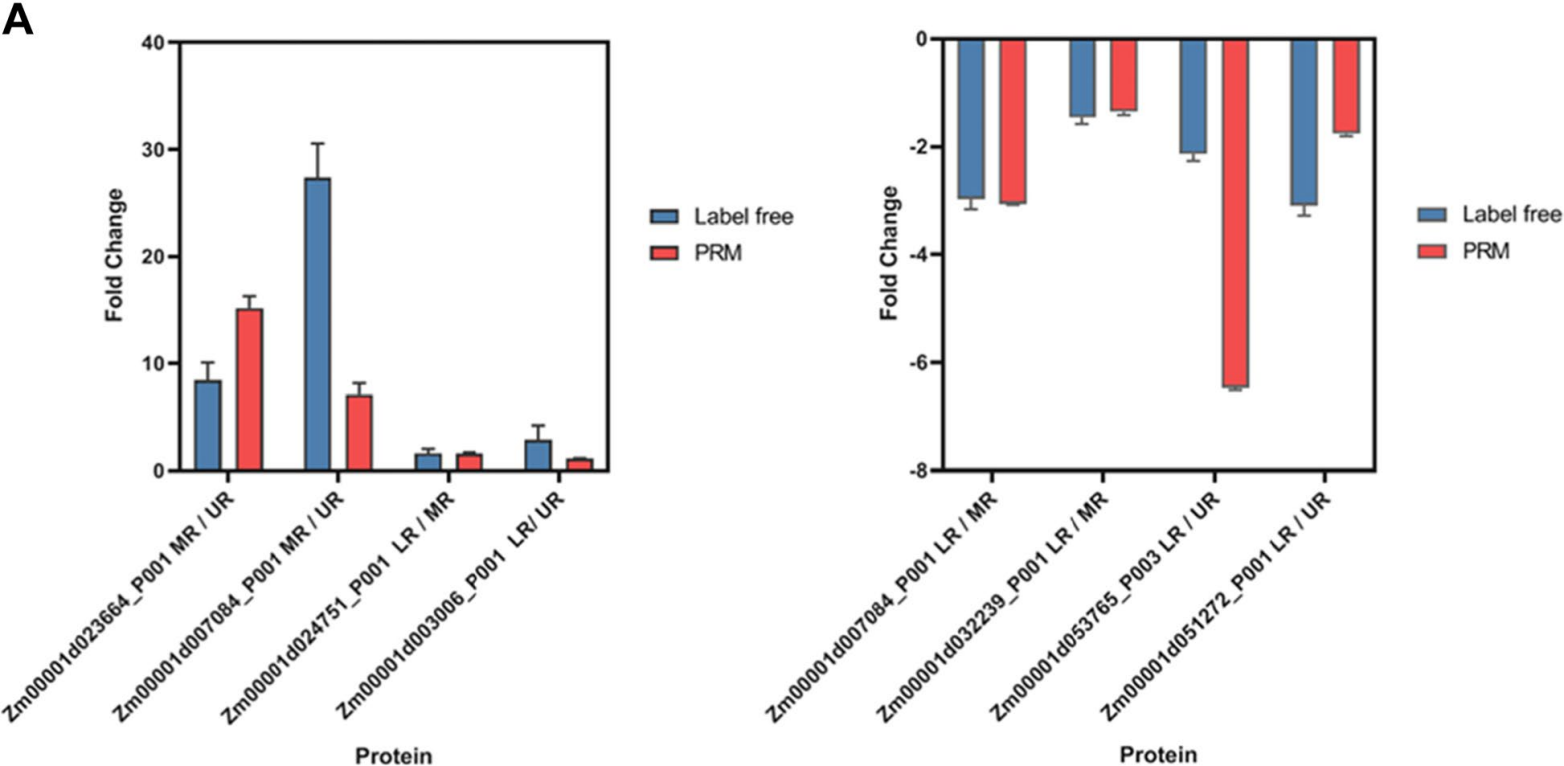
Independent validation of the proteins identified by LFQ-based quantitative proteomics. **a** Parallel reaction monitoring (PRM) validation of several proteins identified by the LFQ data. Blue, bars represent LFQ-based protein abundance values, while red, bars represent PRM protein abundance values

To confirm DAPs identified by the LFQ-based proteomics, we randomly selected 8 proteins from the three comparisons with a unique signature peptide sequence for validation by PRM assay (Fig. 7). Of the 8 DEPs validated by PRM, 4 were up-regulated ABA-responsive protein (Zm00001d023664_P001), histone H2B (Zm00001d007084_P001), peroxidase (Zm00001d024751_P001), and aquaporin PIP2-5 (Zm00001d003006_P001), and 4 was down-regulated histone H2B (Zm00001d007084), HMG-Y-related protein A (Zm00001d032239_P001), V-type proton ATPase subunit c2 (Zm00001d053765_P003) and ascorbate-specific transmembrane electron transporter 1 (Zm00001d051272_P001), Overall, most PRM results showed a good correlation with the corresponding LFQ data.

### Correlation analysis of protein expression and mRNA by quantitative real time polymerase chain reaction (qPCR)

**Fig. 8:**
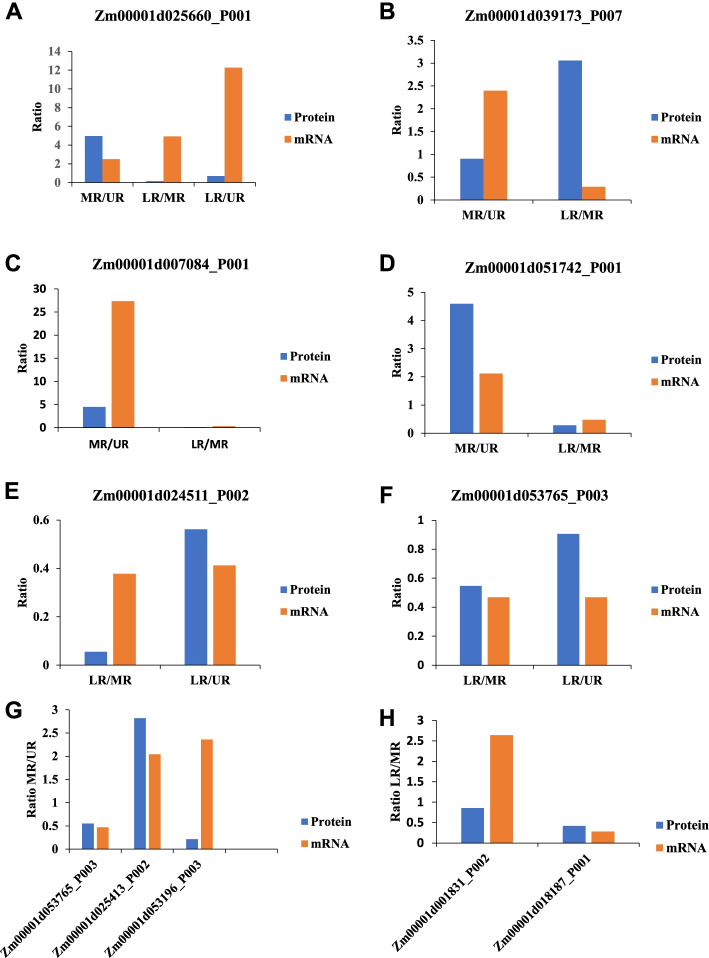
Real-time quantitative PCR analysis. qPCR results of mRNA expression levels of ten proteins randomly selected from LFQ data set. **a-f** mRNA expression levels of six proteins randomly selected from MR/UR, LR/MR and LR/UR. comparison. **g-h** mRNA expression levels of three proteins specifically increased in abundance in MR/UR, and LR/MR respectively. The blue bar and line indicate the protein abundance determined by LFQ and orange bar shows relate expression of mRNA determined by qPCR. All data are presented as mean ± SD (*n* = 3)

Real time PCR results are good for complementing protein level results. Therefore in order to evaluate the correlation between mRNA and protein levels, we analyzed the relative expression pattern of genes encoding ten representative proteins identified by LFQ data with qPCR method (See methods section and Fig. 8). The selected proteins played a role in proton-transporting ATPase activity, glycolysis, regulation of versicular transport, and protein translation. Figure. 8, showed that positive trend correlations between protein and mRNA expression levels were detected for Zm00001d007084_P001, Zm00001d051742_P001, Zm00001d053765_P003, Zm00001d018187_P001, Zm00001d053765_P003, and Zm00001d025413_P002 indicating that abundance of these proteins is likely regulated at the transcriptional level. However, we noticed that the transcript of the Zm00001d025660_P001 in LR/UR, Zm00001d039173_P007 and Zm00001d001831_P002 in or LR/MR, and Zm00001d053196_P003 in MR/UR did not correlate with their protein abundance. Together, more proteins correlated to their corresponding transcript level while the difference in the transcript and protein levels in some of the genes evaluated is probably due to post-translational modifications.

## Discussion

In this study, we investigated the proteome dynamics associated with enhanced maize root water uptake in different root zones (UR, MR, and LR) using a label-free quantitative analysis method. The results of water potential in the different soil layers indicated a significant difference among the root zones, and MR had significantly more water uptake compared to UR and LR. The LFQ based quantitative proteomic analysis provided broad insight to the post-transcriptional events including protein functions underlaying water uptake in maize root. Here over 400 DAPs associated with root functions and differential water uptake were identified in the different root zones (UR, MR, and LR), (Additional file 4: Table S3). More of these proteins were highly abundant in MR and functionally related with multiple biological process such as chromatin structure and dynamics, ribosomal structures and protein translation, polysaccharide and energy metabolism, inorganic ion transport, intracellular trafficking, and vesicular transport. The abundance of several of these candidate proteins in MR highlighted the molecular features underpinning enhanced root water uptake in maize.

### Histones and ribosomal proteins promoted cell wall re-organization to enhance root water uptake

Recent studies indicates that the root wall permeability contributes to soil water acquisition and radial movement through the root system [46–48]. The action of histones is reported to mediate the transcription of enzymes that modify root wall components to improve permeability [48, 49]. In this study, more eight histones were up-regulated in MR compared to UR and LR, such as, histone H2B, histone H2A histone1a and HMG-Y-related protein A (Zm00001d050368 _P001). Which are involved in chromatin remodeling and participate in diverse aspects of root system development including cell fate, cell proliferation, and differentiation [49, 50]. Additionally, histone acetylation has been shown to control the expression of genes essential for cell elongation, and root zonation [51] which could influence root water uptake. Also, we identified several ribosomal proteins that were highly abundant in MR compared to LR. They include 60S ribosomal protein L22-2 (Zm00001d022463 _P001), ribosomal L28e protein family (Zm00001d009417), 60S ribosomal protein (Zm00001d026254 _P001), 60S ribosomal protein L9 (Zm00001d035902 _P001) and plectin/S10 domain (Zm00001d025413 _P001). GO analysis indicate they mainly play a role in the structural organization of the cytoskeleton and ribosome biogenesis. Similarly, more ribosomal proteins were up-regulated in UR compared to LR. These ribosomal proteins are associated with cell cycle regulation, replication recombination, and repair but were significantly down-regulated in LR. Taken together, the increase in abundance of histones and ribosomal proteins in MR supported the enhanced water uptake in MR possibly by modifying proteins abundance that promotes cell wall reorganization and increased root wall permeability, to allowed for more water acquisition [41–43].

### Common induction of protein involved carbohydrate metabolism and transport in the root zones

Carbohydrate metabolism forms the primary respiratory substrate in the root growth. In the present study, we observed common induction of proteins involved in carbohydrate metabolism in MR/UR, LR/MR and LR/UR respectively. Particularly in MR/UR, these DAPs increased significantly in abundance, among them were sucrose synthase (Zm00001d029087 _P002), heteroglycan glucosidase 1 (Zm00001d019497 _P004), beta-glucosidase 17 (Zm00001d041777 _P001), beta-1,3-glucanase (Zm00001d042140 _P001), and glucan endo-13-beta-glucosidase homolog1 (Zm00001d042143 _P001). Most of these DAPs are glucosidases and previous studies suggest that glucosidases are involved in the depolymerization of glucans and hydrolysis of the polysaccharide [52, 53]. Additionally, the result of GO analysis indicated that they played a role in cell wall and membrane envelope biogenesis and polysaccharide cleavage reactions that generate UDP-glucose and fructose which are important substrate for various metabolic reactions [53]. Therefore, the abundance of glucosidases implied an increase in the hydrolysis of complex polysaccharides [54] which could potentially provide glucose as the substrate for energy production.

Also, we observed the specific abundance of cysteine proteinase inhibitors (Zm00001d012561 _P001, Zm00001d043175 _P003, and Zm00001d038558 _P005) in MR. Studies have shown that cysteine-type endopeptidase inhibitors regulate cysteine proteinase activity typically involved in defense response [55, 56], and their specific role in root water uptake is unclear, however, because of their abundance in MR we suspect they might play a regulatory role in maintaining proteins abundance by regulating proteolysis.

Glycolysis, Oxidative Pentose phosphate pathway (OPPP) and tricarboxylic acid cycle (TCA) provides carbon, reduced cofactors, and ATP for cellular functions [57, 58]. In the three root zones (LR/MR and LR/UR comparisons), we observed that important enzymatic steps in the glycolysis pathway and glucose metabolism were up-regulated. For example, phosphopyruvate hydratase (Zm00001d045431 _P002), glyceraldehyde-3-phosphate dehydrogenase (Zm00001d051001 _P004), pyruvate kinase (Zm00001d001831 _P002) and in the step catalyzed by fructokinase-1 (Zm00001d012173_P001, and Zm00001d042536 _P001) more than one protein showed up-regulation in LR/MR. Also, the observed abundance of triosephosphate isomerases (Zm00001d006221 _P001, Zm00001d008619 _P003, and Zm00001d039865 _P001) further highlight the activation of glycolysis and pentose phosphate pathway [59] which provide essential substrates for other metabolic pathways involving energy production in the different root zones. Similarly, in LR/UR, glucose-6-phosphate 1-dehydrogenase G6PD (Zm00001d025015 _P001) a rate-limiting enzyme of the oxidative pentose-phosphate pathway OPPP was specifically induced, whereas 2-oxoglutarate dehydrogenase E1 component (Zm00001d025240 _P004) involved in TCA cycle was specifically induced in UR and MR respectively. The induction of both pathways represents an alternative route for the dissimilation of carbohydrates for generating reducing power besides glycolysis in UR and MR.

Previous studies have described the role of UDP-glycosyltransferases in the regulation of several metabolic processes during plant growth and development in plants including the synthesis of glycans [60–62]. In the present this study, several proteins belonging to the UDP-glycosyltransferase family, i.e., Cis-zeatin O-glucosyltransferase 1(Zm00001d000237 _P001), glycosyltransferase (Zm00001d011649 _P001), dolichyl-diphosphooligosaccharide-protein glycosyltransferase 48 kDa subunit (Zm00001d005248 _P004), and apyrase 1 (Zm00001d007312 _P001), were uniquely up-regulated in UR and MR. Here, we speculated that the abundance of these glycosyltransferases likely contributed substrate to energy production by monosaccharide activation and interconversions through the transfer of sugar moieties to form complex sugars [61, 62].

Contrastingly, we observed phosphoenolpyruvate carboxylase (Zm00001d020057 _P006) a key enzyme of the TCA cycle, was significant repressed in LR. Nevertheless, the common induction of carbohydrate metabolism in the three roots zones suggests that carbohydrate metabolism and transport is an important biological process for maize root development. However, more carbon conversion occurred in MR and UR compared to LR due to the abundance of enzymes related to OPPP and TCA cycle, which could provide more energy and reducing power to support the enhanced water uptake in MR and UR compared to LR.

### Proteins involved energy metabolism and transport in the root zones

Carbon and energy metabolism plays a critical role in root cellular maintenance, growth, water and ion uptake, and transport [58]. In this study, several proteins involved in energy metabolism increased significantly in abundance in MR/UR. They include succinate--CoA ligase [ADP-forming] subunit alpha (Zm00001d006667 _P001), succinate dehydrogenase1 (Zm00001d018758 _P004), aldehyde dehydrogenase 2 (Zm00001d051754 _P002), and mitochondrial phosphate carrier protein 3 (Zm00001d018187 _P001) Previous studies have shown that the presence of TCA cycle enzymes is critical for cellular energy metabolism and plays a key role in root development [43, 63]. Here, we conclude that the abundance these TCA cycle enzymes in MR enhanced ATP synthesis and NADH/NADPH including other TCA cycle intermediates required for energy metabolism to support diverse root functions in MR including enhanced water uptake.

High ATP production required for enhanced water uptake in MR was further supported by abundance of mitochondrial electron transport chain that mediates the generation of electron gradients for ATP production. Such as cytochrome B5 isoform D (Zm00001d041963 _P001), cytochrome b-c1 complex subunit Rieske (Zm00001d016619 _P002), cytochrome c1 1 heme protein mitochondrial (Zm00001d049959 _P005). These proteins are involved in coupling electron transfer from organic substrates onto molecular oxygen, and the transfer of proton across the inner mitochondrial membrane [64]. The resulting proton gradient is used by the ATP synthase complex for ATP production which can be utilized in MR for water and nutrient uptake and transport with the root system. In addition, we found that malate dehydrogenase (MDH) (Zm00001d032187 _P001) involved in the interconversion between malate and oxaloacetate (OAA) and supplies NAD [65], and NADH dehydrogenase [ubiquinone] 1 alpha subcomplex subunit 9 mitochondrial (Zm00001d018479 _P001) involved in the mitochondrial electron transport chain and proton translocation across the mitochondrial membrane [66]. These results are consistent with previous studies [58] which suggest that water/nutrient uptake and transport through by roots cells layers require cellular energy and reducing power therefore abundance of ATP producing enzymes aligns with the increased rates of water uptake in MR [67]. Furthermore, we suspect that MR might have improved nutrients uptake considering the abundance of ATP producing enzymes and the role of plasma membrane (PM) H + -ATPase that uses ATP to pump H+ towards the apoplast to generate proton motive force which provides energy to drive nutrient diffusion and its distribution through PM [68]. However, more experiments are needed to test this hypothesis.

Previous studies indicate the membrane-bound proteins and ion transporters play crucial role in the transport of water and nutrient in the root system [69, 70]. Specifically, ion transporters that use ATP hydrolysis to drive protons transport across cell membranes to energize water and nutrient transportation. In the present study, we found some membrane-bound proteins and ion transporters were specifically induced in UR. For instance, the ATP synthase epsilon chain (Zm00001d044441 _P002) with function related to proton-transporting ATP synthase activity, thioesterase (Zm00001d048583 _P001) involved in oxidative phosphorylation and mitochondrial transmembrane transport; and NADH ubiquinone reductase (Zm00001d049597 _P003), a mitochondrion inner membrane protein that catalyzes the reduction of NADH to NAD+. These enzymes likely contributed to energy production in UR and could support it moderate water uptake observed in UR. Similarly, in LR, we identified unique proteins that were associated with cellular energy metabolism, i.e., ATP synthase delta chain (Zm00001d051595 _P004), ATP citrate synthase (Zm00001d041593_P001), including electron transport chain: H (+)-exporting diphosphatase (Zm00001d046591 _P003), cytochrome c oxidase polypeptide Vb (Zm00001d044459 _P008) and cytokinin dehydrogenase (Zm00001d039520 _P001).

Interestingly, we detected the significant repression of mitochondrial phosphate carrier protein 3 (Zm00001d018187 _P001) and phosphoenolpyruvate carboxylase3 in the LR. GO, and COG analysis revealed that the mitochondrial phosphate carrier protein is involved in phosphate ion transmembrane transport. Meanwhile, phosphoenolpyruvate carboxylase catalyzes the formation of oxaloacetate, an essential substrate for the TCA cycle. Additionally, hemoglobin1 (Zm00001d048020 _P001), V-type proton ATPase subunit G (Zm00001d005253 _P002), and V-ATPase 69 kDa subunit (Zm00001d053765 _P003) decreased significant in abundance in LR, opposite to their abundance in UR. Here, the significant downregulation of both proteins suggests that energy conversion and electron transport across membranes may be limited and could result in low water uptake in LR.

### Water channels and inorganic ion transmembrane transport proteins in the root zones

The main pathways of root water uptake are the apoplastic and cell-to-cell pathways [71]. The apoplastic pathway consists of radial water flow through the intercellular space and cell wall, while the cell-to-cell pathway includes water flow across cytoplasm and membranes of neighboring cells [71, 72]. Both pathways contribute to the total water flow across the root tissue [71–74]. Aquaporins are water channel proteins that regulate water transport in cells. They are localized in various cell membrane compartments of plant cells [73, 74] and form aqueous pores that selectively allow passive transport of solutes across the various cell membrane. The abundance and activities of aquaporin affect root hydraulic properties, cell permeability, and whole root tissue water uptake [73–77]. In the present study, we detected two water channels proteins: aquaporin PIP1-3/PIP1-4 (Zm00001d051403 _P001) and aquaporin PIP1-5 (Zm00001d051872 _P001) in MR/UR. The aquaporin PIP1-3/PIP1-4 was induced in both MR and LR, but in MR it was up-regulated and repressed in LR. Similarly, aquaporin PIP1-5 was up-regulated in MR, but downregulated in UR and was not abundant in LR. The abundance of the aquaporins in the MR aligns with the enhanced water uptake observed in this zone (Fig. 1). The specific abundance of these proteins was confirmed by PRM analysis.

Also, we detected other aquaporin isoforms that were up-regulated in LR and UR, for example, aquaporin PIP2-3 (Zm00001d051174 _P001) and aquaporin PIP2-5 (Zm00001d003006 _P001). Here, aquaporin PIP2-3 was abundant in all three root zones but was up-regulated more than 3 folds in LR compared to its normal abundance in MR and UR. Meanwhile, aquaporin PIP2-5 (Zm00001d003006 _P001) was up-regulated in LR but was not in UR. These results suggests that the specific up-regulation of aquaporins in each of the three root zones played a positive role in maize root water uptake, however its abundance contributed to increase water uptake in the MR zone [75–77].

Mineral nutrient uptake is accompanied by water absorption [78] and the acquisition of mineral ions from the soil is regulated by water channel proteins and other cellular transporters localized to root plasma membranes [78–84]. Previous studies have shown that some aquaporins transport potassium [78], phosphorus [78], Calcium (Ca) [79], ammonia (NH3) [80–82], urea [81, 82], boric acid [83, 84], and hydrogen peroxide (H2O2) [85]. In the present study, we identified proteins involved in mineral ion transport in the three root zones. Such as, zinc transporter2 (Zm00001d041959 _P001), citrate transporter1 (Zm00001d027667 _P014), sulfurtransferase (Zm00001d031024 _P003), and molybdopterin-molybdenumtransferase (Zm00001d001970 _P001). These proteins were uniquely abundant in MR. Also, several of proteins such as the zinc transporter, sulfurtransferase and molybdopterin-molybdenumtransferase were up-regulated in UR. However, in LR, many of the mineral ion transporters were not differentially abundant, and those that were induced were significantly repressed including ascorbate-specific transmembrane electron transporter1 (Zm00001d051272 _P001), calcium pump2 (Zm00001d028687 _P001), and superoxide dismutase (Zm00001d029170 _P002). The downregulation of mineral ion transporters negatively impacts root ion exchange at the surface of the soil layer [86], and may have contributed to the low water uptake in the LR.

### Protein involved in amino acid uptake and transport in the root zones

Inorganic nitrogen in the form of ammonium and nitrate; and organic nitrogen such as amino acids, peptides, are taken up, distributed, and assimilated by plant roots, by water channels and membrane transport proteins [87, 88]. In the present study, several proteins related to amino acid metabolism and transport were identified. For instance, phenylalanine ammonia-lyase (PAL) (Zm00001d017274 _P001) and amine oxidase (Zm00001d025103 _P001) were significantly up-regulated in MR but down-regulated in UR. The PAL catalyzes phenylalanine deamination to form transcinnamic acid and free ammonia [89]. The amine oxidases catalyze the oxidative deamination of polyamines, which are ubiquitous compounds essential for cell growth and proliferation [90]. Likewise, the hydrolase, carbon-nitrogen family protein (Zm00001d016704 _P001), and protein NRT1/ PTR family 5.10 (Zm00001d038334 _P001) where specifically abundant MR. Hydrolase-carbon-nitrogen family protein have been shown to cleave various nitriles producing carboxylic acids and their corresponding amides [91, 92], thereby contributing to free inorganic nitrogen sources for root uptake. Whereas NRT1/ PTR, a proton-dependent oligopeptide transporter which belongs to the major facilitator superfamily are involved in the uptake of small peptides, nitrate uptake, and histidine transport [93, 94]. Taken together, the up-regulation of these proteins indicates an efficient uptake of inorganic nitrogen and its metabolism and transport occurred in the MR.

Of interest is the repression of glutamine synthetase (Zm00001d028260 _P003), glycine cleavage system H protein (Zm00001d015378 _P001), and chorismate mutase 2, cytosolic cm2 (Zm00001d015509 _P002) among proteins identified in MR/UR. For example, glutamine synthetase plays an essential role in nitrogen metabolism by catalyzing the condensation of glutamate and ammonia to form glutamine [95]. The glycine cleavage system H protein shuttles methylamine group of glycine from the P-protein (glycine dehydrogenase) to the T-protein (aminomethyltransferase) [96], and chorismate mutase 2 plays a role in protein synthesis and acts as a precursor of a wide range of aromatic amino acids such as phenylalanine and tyrosine [97]. The repression of these proteins in the MR suggests a reduction of cellular amino acid metabolic activities.

Contrastingly, glutamate synthase 1 [NADH] chloroplastic (Zm00001d043845 _P031) was up-regulated in LR compared to the abundance of its isoform in UR and MR respectively. We noted that additionally, we observed the abundance of anthranilate synthase (Zm00001d028536 _P002), aspartate aminotransferase (Zm00001d016198 _P004), and ketol-acid reductoisomerase (Zm00001d044035 _P001), these proteins were up-regulated in LR and are functionally related to nitrogen metabolic precursors and nitrogen-transport process [98, 99]. Other amino acid biosynthetic proteins specific to LR, were homoserine kinase (Zm00001d018555 _P001), leucine aminopeptidase 2 chloroplastic (Zm00001d016551 _P002), and argininosuccinate lyase chloroplastic (Zm00001d047667 _P001), showed a significant increase in abundance and possibly played a positive role in organic nitrogen uptake and metabolism in the LR zone.

Similar to LR, most of the amino acid biosynthesis and transport-related proteins increased specifically in abundance in the UR, such as lysine histidine trans-porter 2 (Zm00001d035157 _P002), glycine cleavage system H protein, glutamate dehydrogenase (Zm00001d025984 _P002), and phenylalanine ammonia-lyase (Zm00001d033286 _P001). Taken together, DAPs related to inorganic nitrogen [100], could contribute solute uptake in this MR zone. While most of the DAPs involved amino acid metabolism in the UR and LR zones could be play a role in organic nitrogen assimilation. Nevertheless, more studies are required to evaluate nitrogen content of the different root zones.

### Protein involved intracellular trafficking, secretion, and vesicular transport in the root zones

Water uptake from the soil and its radial transport toward the vascular tissues is achieved by membrane transport proteins, including water channels and other specialized transmembrane transporters [100]. Membrane transport proteins must be properly targeted and tethered to the plasma membrane to transport secreted proteins [101]. Therefore, identifying membrane transport proteins associated with trafficking in different maize root zones could help shed light on the molecular mechanism underlaying enhanced water uptake. Earlier studies identified SNAREs, vesicle coat proteins, GTPases, and tethering factors as the major protein involved in intracellular trafficking and vesicular transport [102–104]. In the present study proteins associated with intracellular trafficking and vesicular transport were up-regulated in MR compared to UR and LR zones. For example, we noted major proteins syntaxin-132 (Zm00001d041716 _P006) associated with SNAP receptor activity and vesicle docking was significantly downregulated in LR and but up-regulated in MR and UR zone. Whereas, the ADP-ribosylation factor A1B (Zm00001d008295 _P001), thaumatin-like protein (Zm00001d030694 _P002), coatomer subunit delta (Zm00001d023943 _P007), exocyst complex component SEC5 (Zm00001d025398 _P012) and exocyst complex component SEC10 and (Zm00001d041138 _P003). Of interest are the proteins that constitute the exocyst complex, the exocyst complex component SEC5 was abundant in MR and LR but exocyst complex component SEC10 increased in abundance specifically in MR. These proteins are directly involved golgi to the plasma membrane transport and associated with the docking of exocytic vesicles to the plasma membrane [105]. Additionally, we observed specific abundance of dynamin-like protein ARC5 (Zm00001d023583 _P004), and GTP-binding protein homolog1 (Zm00001d034949 _P001) in MR. Dynamin-like protein ARC5 is also a member of the GTPases protein superfamily, which are involved in the budding and scission of nascent cargo vesicles from one cellular compartment to another [106]. Proteins involved in mitochondrial solute exchange with the sounding cells, intermembrane translocation between the ER, and the Golgi apparatus, reticulation, and vesicular transport, were also abundant in MR and UR respectively. For example, we detected positive induction of mitochondrial import inner membrane translocase subunit Tim9 (Zm00001d027342 _P002), vacuolar protein sorting-associated protein 35 (Zm00001d034492 _P017), and reticulon-like protein (Zm00001d039316 _P004), their abundance supported the transport of vesicles from plasm membrane to vacuoles [106–109]. Taken together, these results indicate that the abundance of vesicular transport proteins in the MR zone contributed to its enhanced water up-take rates. Whereas their low abundance and repression could explain the lower rates of water uptake in UR and LR zones.

## Conclusion

**Fig. 9:**
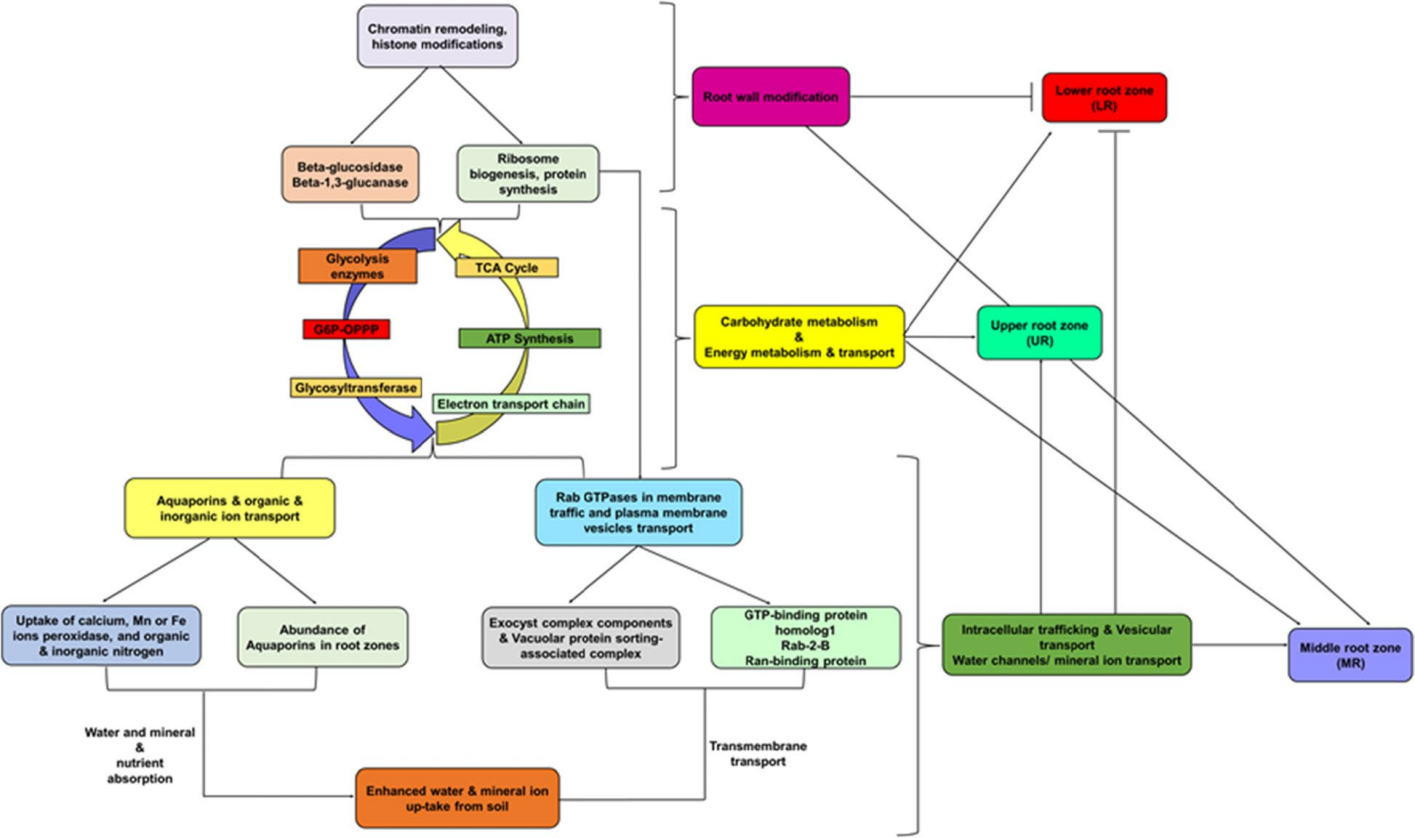
Proposed model for enhanced water uptake in maize root zones. The model shows the potential mechanism of water uptake in maize root and highlighted specific proteins underlaying water uptake

To our knowledge this study is the first to apply LFQ-based quantitative proteomics to characterize protein profiles in different root zones (UR, MR, and LR) that are related to water uptake in maize under smart irrigation. Analysis of soil water potential within each root zone indicated that MR had more water uptake compared to UR and LR. LFQ examination of protein abundance pattern revealed profile of the proteins associated with root water uptake at UR, MR, and LR respectively. GO and KEGG analysis highlighted common and significant differences in the protein function underlaying water uptake in each root zone. For example, proteins associated with carbohydrate metabolism and energy production were common to the root zones and possibly necessary for normal water uptake. However, abundance of proteins involved in chromatin structures and dynamics, ribosomal protein synthesis, electron transport chain, water channels, inorganic ion transport and transmembrane and vesicular transporters played key role in enhancing root water uptake observed in MR. Based on our results we proposed a model in Fig. 9. In the model, we highlighted the specific abundance of the enzymes and proteins associated to cell wall permeability, carbon and energy metabolism, water channels proteins and vesicular transport potentially underpinned enhanced root water uptake in maize. Overall, the proteins and their abundance profile reported in this study provided an insight into the mechanisms of enhanced root water uptake in maize.

## Materials and methods

### Plant material and pot growth system

Materials and the maize variety Anyu308 (*Zea mays* L.) was chosen for this study and was grown in the greenhouse of the Institute of Cotton Research of the Chinese Academy of Agricultural Sciences (ICR CAAS) in Anyang, Henan, China (longitude 36°06 N and latitude 114°21 E). The experimental research on the maize plants including sample collection was performed according to institutional guidelines under the local legislation. The plant growth condition was set to a photoperiod of 14 h, day/night, with a temperature of 25 °C/22 °C, relative humidity of 60%, and light intensity of 500 μmol m-2 s-1. The multi-depth soil moisture meter (Smart Moisture), an intelligent internet soil moisture sensor data logger, was used to monitor the moisture changes in re-al-time at various soil depths. The plants were grown triplicates in 10 cm diameter polyvinyl chloride (PVC) pots and filled with a mixture of silt and quartz sand, sieved to a particle diameter of < 1 mm and density of 1.5 g cm-3. The smart moisture was placed in the middle of the pots, and the soil surface surrounding the smart moisture was covered with a layer of black film to eliminate the soil evaporation. Several holes with a diameter of 1.2 mm were drilled at the bottom and sides of the pots to allow for water drainage and irrigation. Maize seeds were germinated on moist filter paper for 48 h, and three maize seedlings were planted in each container. The total number of pots was 6.

### Water uptake measurements

The plants were grown for 90 days with regular irrigation when the corn root system is fully developed, irrigation was stopped for 24 h, and water was allowed to drained to achieve uniform soil moisture conditions. After 24 h, the soil moisture levels of all soil layers 10 cm, 20 cm, 30 cm, 40 cm, 50 cm, 60 cm, respectively was taken and used to determine the maximum field water holding capacity. Subsequent irrigation was scheduled automatically when the soil moisture content is at 70% of the maximum field water holding capacity; this was termed the irrigation cycle, which takes be-tween 3 -4 days. The amount of water used by plants in one irrigation cycle was determined by measuring soil moisture content (FC10, FC20, FC30, FC40, FC50, and FC60, respectively) at field water capacity in each soil layer (10 cm, 20 cm, 30 cm, 40 cm, 50 cm, 60 cm, respectively) using the Smart Moisture meter until irrigation compensation point was reached. Afterward, the final reading of soil water content at each soil layer (RP10, RP20, RP30, RP40, RP50, and RP60, respectively) was taken. The smart moisture meter took a total of 4 reading cycles from the field holding to the irrigation compensation point. The root water uptake was indirectly determined as the absolute difference in water content of soil in each layer measured between the field holding capacity and the irrigation compensation point (△M10, △M20, △M30, △M40, △M50, △M60).

$$\mathrm{Irrigation}\;\mathrm{volume}\;=\;\left[\mathrm{field}\;\mathrm{water}\;\mathrm{holding}\;\mathrm{capacity}\right]/\left[\mathrm{current}\;\mathrm{water}\;\mathrm{holding}\;\mathrm{capacity}\right].$$

Three representative plants from each replicate were sampled, the roots were washed, the length measured, then based on the soil layer divided the into three distinct roots zones, i.e., Upper root (0 cm-20 cm), Middle root (20 cm-40 cm), and Lower root (40 cm-60 cm). Each distinct zone was weighed and used for label-free quantitative proteomics (LFQ) experiment.

### Protein extraction

Protein extraction was performed using three individual biological replicates for each sample. Briefly, 1 g of leaf tissue was weighed, homogenized by grinding in liquid nitrogen, lysed with lysis buffer (Tris–HCl [pH 8], 8 M urea, 0.2% SDS, 1× phosphoprotein protease inhibitor complex), ultrasonicated on ice for 5 min, and centrifuged (12,000×g, 20 min, 4 °C). The supernatant was transferred to a clean tube. Proteins were precipitated in pre-cooled acetone at − 20 °C for 2 h, washed 2× in 75% ethanol, and resolved in lysis buffer. Protein concentration in the lysate was estimated using bicinchoninic acid (BCA) protein assay kit (Beyotime Institute of Biotechnology, China). The rest of the lysate was frozen at − 80 °C until use.

### Protein digestion

Filter-aided sample preparation (FASP) method was used for the on-filter digestion of proteins [110]. Here protein concentrates (300 μg) in an ultrafiltration filtrate tube (30 kDa cut-off, Sartorius, Gottingen, Germany) was mixed with 200 μL UA buffer (8 M urea, 150 mM Tris-HCl, pH 8.0) and centrifuged at 14,000 g at 20°Χ for 30 min and an additional washing step with 200 μL of UA buffer. Then 100ul of 50 mM iodoacetamide in UA buffer were subsequently added to the filter to block the reduced cysteine residues, and the samples were then incubated for 30 min at room temperature in the dark, followed by centrifugation at 14,000×g for 30 min. The filters were washed three times with 100 μL of UA buffer and centrifuged at 14,000×g for 30 min after each washing step. The protein suspensions were then digested with 40 μL of trypsin (Promega, Madison, WI, USA) buffer (6 μg trypsin in 40 μL dissolution buffer) at 37 °C for 18 h. Finally, the filter unit was transferred to a new tube, added 40 μL dissolution buffer, and centrifuged at 14,000×g for 30 min. The resulting peptides were collected as a filtrate, and the peptide concentration was analyzed at OD280.

### LC-MS/MS analysis and data analysis

The peptide samples were analyzed using the Easy-nLC nanoflow HPLC system connected to Orbitrap Fusion mass spectrometer (Thermo Fisher Scientific, San Jose, CA, USA). A total of 1 μg of each sample was loaded onto the Thermo Scientific EASY column (two columns) using an autosampler at a flow rate of 200 nL/min. The sequential separation of peptides on Thermo Scientific EASY trap column (100 μm × 2 cm, 5 μm, 100A, C18) and analytical column (75 μm × 25 cm, 5 μm, 100 Å, C18) was accomplished using a segmented 1 h gradient from 5 to 28% Solvent B (0.1% formic acid in 100% ACN) for 40 min, followed by 28-90% Solvent B for 2 min and then 90% Solvent B for 18 min. The column was re-equilibrated to its initial highly aqueous solvent composition before each analysis. The mass spectrometer was operated in positive ion mode, and MS spectra were acquired over a range of 375-1500 m/z. The MS scan and MS/MS scan resolving powers at 200 m/z for the Orbitrap Fusion were set as 120,000 and 50,000, respectively. Data Dependent Mode is Top Speed, Cycle Time is 3 s, and ions were fragmented through higher energy collisional dissociation. The maximum ion injection times were set at 50 ms for the survey scan and 105 ms for the MS/MS scans, and the automatic gain control target values for Master scan modes were set to 4e5, and MS/MS was 1e5. The dynamic exclusion duration was the 40s. The raw files were analyzed using the Maxquant 1.6.5.0 software (Thermo Fisher Scientific) [111]. Search for the fragmentation spectra against the Zea_mays_AGPv4_pep.fast (131,496 sequences). Search parameters were according to [111] Protein abundance was estimated using the LFQ quantification method. The MS data were deposited in the ProteomeXchange Consortium database via the PRIDE partner repository, with the PXD identifier (PXD026269).

### Bioinformatics

Maxquant 1.6.5.0 Protein quantitation values were exported for further analysis in Excel. Proteins of *p*-values < 0.05 by Student t-test and a fold-change of > 2 or < 0.5 in abundance between any two groups were considered significant. GO annotation for the differentially abundant proteins (DAPs) was derived using Blast2GO software v 4.1.9 [112]. Pathway analyses were extracted using the Search pathway tool in the KEGG Mapper platform (http://www.genome.jp/kegg/mapper.html). Fisher’s exact test was used to per-form the pathway enrichment statistics; *p* < 0.05 was set as the threshold used for enrichment analysis of KEGG pathways [113]. The search tool for the retrieval of interacting genes/proteins (STRING) database for physical and functional interaction prediction was used to analyze the protein-protein interaction (PPI) network [114]. The graphical visualization and analysis of the interaction network were performed in Cytoscape 3.2 [115].

### Liquid chromatography-parallel reaction monitoring/mass spectrometry (LC-PRM/MS) and data analysis

The LC-PRM/MS analysis was used to confirm the abundance of the proteins obtained by LFQ-based quantitative proteomics analysis. The peptide information suitable for PRM were selected and was imported into the Xcalibur soft-ware program for the PRM setup. Here, 2-μg peptide from each sample was taken for LC-PRM/MS analysis. After sample loading, the chromatographic separation was performed using a Thermo Scientific EASY-nLC nano-HPLC system. The following buffer was used: solution A, 0.1% formic acid aqueous solution, solution B, a mixed solution of 0.1% formic acid, acetonitrile, and water (95% 12 of acetonitrile). First, the column was equilibrated with 95% solution A. The sample was injected into a Trap column (100 μm × 20 mm, 5 μm-C18, Dr. Maisch GmbH) and then subjected to gradient separation through a chromatography column (75 μm × 150 mm, 3 μm-C18, Dr. Maisch GmbH) at a flow rate of 300 nL/min. Afterwards the peptides were separated and subjected to targeted PRM/MS using a Q-Exactive Plus mass spectrometer (Thermo Scientific). The obtained PRM data was analyzed using the Skyline 4.1 software program.

### RNA extraction and quantitative real-time PCR (qPCR)

Total RNA was extracted from each maize root sample using TRIZOL reagent (Invitrogen, Carlsbad, CA, USA). The RNA quantity and quality were determined with a NanoDrop 2000 spectrophotometer (Thermo, USA) according to the manufacturer’s instructions. The cDNA was synthesized from the RNA using the PrimeScript Reverse Transcriptase Kit (Takara, Dalian, China) for quantitative real-time polymerase chain reaction (qPCR). The gene-specific primers for the qPCR are listed in Additional file 10: Table S9. The PCR condition is as follows, 40 cycles of 95 °C for 15 s and 60 °C for 30 s). The gene expression levels were quantified relative to the maize Ubi gene with 2–ΔΔCT method [116]. Each reaction was performed in three replicates.

## Supplementary Information

**Additional file 1: Table S1.** List of proteins identified by LFQ analysis.

**Additional file 2: Table S2.** List of peptides identified by LFQ.

**Additional file 3: Figure S1.** Correlation plots of three biological replicates of all samples. R2 values are consistent and are noticeably higher between the replicates.

**Additional file 4: Table S3.** List of differentially expressed proteins.

**Additional file 5: Table S4.** List of GO terms assigned to DAPs identified in the three root zones.

**Additional file 6: Table S5.** List of COG for differentially expressed proteins.

**Additional file 7: Table S6.** List of KEGG terms for differentially expressed proteins.

**Additional file 8: Table S7.** List of protein-protein interaction network.

**Additional file 9: Table S8.** List of candidate proteins associated with enhanced root water uptake in maize.

**Additional file 10: Table S9.** The list of gene-specific primers selected for qRT-PCR analysis.

## Data Availability

The mass spectrometry proteomics data have been deposited to the ProteomeXchange Consortium (http://proteomecentral.proteomexchange.org) via the iProX partner repository with the dataset identifier PXD026269.
