# Supplementary figures and images for: Label-free quantitative proteomics of maize roots from different root zones provides insight into proteins associated with enhance water uptake
Source: BMC Genomics. 2022 Mar 6;23:184. doi: 10.1186/s12864-022-08394-y (PMC8898408; doi:10.1186/s12864-022-08394-y)

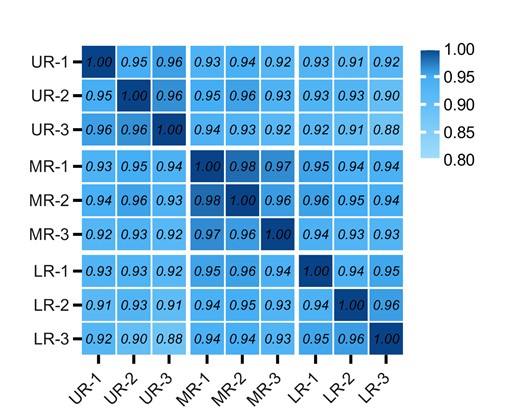

Supplement: Supplementary file 3 — Additional file 3: Figure S1. Correlation plots of three biological replicates of all samples. R2 values are consistent and are noticeably higher between the replicates. [file 12864_2022_8394_MOESM3_ESM.jpg]
